# Rethink cities to promote an active lifestyle and tackle climate change: best practices from the OECD

**DOI:** 10.1093/eurpub/ckac129.221

**Published:** 2022-10-25

**Authors:** M Cecchini, A Piatrova, A Aldea, A Lerouge, J Cheatley

**Affiliations:** OECD, Paris, France

## Abstract

**Background:**

City design can have major health and environmental implications. The overall layout of cities influences air quality, as urban sprawl can encourage the use of motorised vehicles and decrease active travelling. Furthermore, lack of trees has a negative impact on particulate matter levels and contributes to the urban heat island effect. There is a growing interest for creating an urban environment conducive of healthier lifestyles.

**Methods:**

The analysis uses the five OECD criteria to assess best practices in public health - Effectiveness, Efficiency, Equity, Evidence-base, and Extent of coverage - to carry out a systematic assessment of selected candidate best practices to improve the public health potential of cities. The impact of scaling up these interventions within and across countries is evaluated by using the OECD SPHeP-NCD microsimulation model.

**Results:**

Interventions such as Superblocks in Barcelona, which reshapes the city layout to make them more people centric and less vehicles reliant, or Cycle Superhighways from Denmark, which develops cycling networks, have a the potential to avoid a significant number of chronic diseases by promoting an active lifestyle and decreasing transport-related pollution. Such interventions can also decrease healthcare expenditure and, if well designed, health disparities.

**Discussion:**

While many urban design interventions are a good investment for countries and, in general, there is good support, a number of implementation hurdles exist. First, extent of coverage is still relatively limited across European countries. Second, changes take time to be implemented and some of the health impact materializes in the longer-term. Third, the implementation of such interventions is generally competence of other authorities, other than the health authorities. Building strong multi-stakeholder approaches and making a strong case for such investments can promote change.

